# The role of mindfulness in quality of life of persons with spinal cord injury: a cross-sectional study

**DOI:** 10.1186/s12955-022-02059-w

**Published:** 2022-10-31

**Authors:** Muna Bhattarai, Susan Miller Smedema, William T. Hoyt, Malachy Bishop

**Affiliations:** 1 School of Nursing, Texas A&M University, 8447 Riverside Pkwy, 77807-3260 Bryan, TX USA; 2 Department of Rehabilitation Psychology & Special Education, University of Wisconsin–Madison, Madison, WI USA; 3 Department of Counseling Psychology, University of Wisconsin–Madison, Madison, WI USA

**Keywords:** Spinal cord injury, Quality of life, Mindfulness, Pain, Functional limitation

## Abstract

**Background:**

Quality of life is considered the most overarching psychosocial adaptation outcome following the rehabilitation of persons with spinal cord injury. Literature suggests that the quality of life of persons with spinal cord injury is determined by many personal and psychological factors, including mindfulness. This study aimed to identify the direct and indirect effect of mindfulness on the quality of life of persons living with spinal cord injury.

**Methods:**

Participants consisted of 231 members of three spinal cord injury organizations in the United States: United Spinal Association, North American Spinal Cord Injury Consortium, and Paralyzed Veterans of America-Wisconsin Chapter. The participants completed a set of standardized self-report questionnaires in an online Qualtrics survey. A hierarchical regression analysis was performed to identify the contribution of mindfulness to quality of life, controlling for sociodemographic and injury-related factors. A serial mediation analysis was performed to examine the indirect effect of mindfulness on quality of life.

**Results:**

In the hierarchical regression analysis, sociodemographic and injury-related factors (i.e., age, gender, race, marital status, education, employment, level and completeness of injury, comorbidities, frequency of hospitalization, pain intensity, and functional limitation) and mindfulness explained 59% variance on quality of life of the participants with spinal cord injury. Mindfulness uniquely contributed to the higher quality of life above and beyond sociodemographic and injury-related variables. In the serial mediation analysis, pain and functional limitation did not significantly mediate the relationship between mindfulness and quality of life. However, the indirect effects of mindfulness on functional limitation and quality of life through pain were significant.

**Conclusion:**

The findings underscore the vital role of mindfulness in improving the quality of life of persons with spinal cord injury. Implications of these findings for future research and clinical practice are discussed.

## Introduction

Quality of life (QOL) is considered to be the most overarching psychosocial adaptation outcome for people with chronic illnesses and disabilities (CIDs) [1]. QOL is a multidimensional construct that is composed of the subjective and objective evaluation of multiple life domains, such as physical, mental, social, and financial aspects of life [2, 3]. Many individuals with CIDs, including spinal cord injury (SCI), report low QOL compared to the general population [4]. SCI, a chronic neurological condition, is one of the most common causes of long-term disability [5]. Because SCI is associated with the risk of developing various secondary complications, including physical and psychosocial problems, persons with SCI (PwSCI) are likely to experience poor adaptation, including low QOL [4]. A rich body of literature shows that many personal and environmental factors play a significant role in determining the QOL of PwSCI [5–8].

Some evidence suggests that sociodemographic and SCI-related factors significantly influence QOL or wellbeing in PwSCI. The individuals’ age [4, 6, 9, 10], employment status [11–13], education status [14, 15], marital status, and gender [16] have been reported to impact QOL among PwSCI. Similarly, level of injury [6, 10, 17], pain intensity [18, 19], hospitalization, and functional limitation [4, 6, 20, 21] were found to be associated with QOL. However, the effect of some sociodemographic and injury-related factors on QOL is still inconclusive [4, 22, 23]. SCI-related pain not only affects QOL but also results in functional limitation. The presence of pain, and in particular, the location of the pain, is associated with functional dependence. For instance, if individuals with SCI have pain in the upper extremities, it may impact their ability to use a wheelchair and perform basic activities of daily living [24]. Taken together, some sociodemographic and injury-related variables are likely to contribute to QOL in PwSCI; however, other psychological factors also affect adaptation outcomes.

### Mindfulness

In addition to sociodemographic and injury-related factors, psychological resources such as mindfulness are key determinants of psychosocial adaptation in many individuals [25–31]. Livneh’s psychosocial adaptation to chronic illness and disabilities model posits psychosocial adaptation as a dynamic and long-term process resulting from the interaction among demographics, health-related, psychological, and social factors [1, 32]. Mindfulness, a crucial psychological factor, is an emerging concept in positive psychology and refers to increased attention to open and receptive awareness of one’s current experience or reality [25]. Kabat-Zinn (2003) defines mindfulness as a process of cultivating awareness by paying attention to the present moment as non-reactively, non-judgmentally, and openheartedly as possible. Mindfulness or mindfulness-based interventions have been consistently identified as a significant positive contributor to favorable psychosocial adaptation outcomes (e.g., QOL, wellbeing) in people with various CIDs, including those with multiple sclerosis, cancer, and SCI [26, 27, 30, 31, 33]. A systematic review of five studies reported the efficacy of a mindfulness-based intervention in reducing anxiety and depression and improving the QOL in PwSCI [26]. Davis and Hayes [33] stress that mindfulness enhances emotional regulation and increases one’s attentional skills and ability to manage distraction. Mindful persons tend to be aware of the present moment and their emotional state, which helps them regulate their emotions and experience more positive emotions [34].

Mindfulness directly contributes to higher QOL and is also likely to mitigate the adverse effects of SCI-related secondary complications (e.g., pain, functional limitation) on QOL. Literature suggests that pain and functional limitation are improved with mindfulness or mindfulness-based interventions [27, 28, 35–37]. Individuals with a high level of mindfulness gain increased moment-to-moment awareness of their physical and psychological experiences related to disability conditions [31]. Hearn and Finlay [27] reported the effectiveness of online mindfulness training on pain reduction in PwSCI. Mindfulness training may minimize the perceived barriers to pain management and increase pain acceptance without judging it as an unpleasant experience, likely decreasing pain perception [27]. It appears that individuals who are aware of pain do not attempt to avoid it, focus on living life to the fullest with acceptance of it, and perceive pain as less bothersome [35]. Even when a person has pain, being mindful of pain may help them maintain QOL despite the presence of the impacts of such chronic pain.

The contribution of mindfulness to functional independence has not been studied extensively among PwSCI. A previous study revealed a negative relationship between mindfulness and functional limitation in people with breast cancer. A mindfulness-based intervention significantly decreased functional limitation in post-intervention and follow-up [36]. The researchers claimed that mindfulness allows one to focus on the present moment without emotionally reacting to it and helps recognize and control the triggering and perpetuating factors related to functional limitation, thereby improving functional independence, and consequently, QOL [36].

Despite some promising evidence on the benefits of mindfulness, only a few studies have examined the contribution of mindfulness to QOL in PwSCI [26, 27]. Chronic pain and functional limitation are prominent secondary complications that profoundly influence the QOL of PwSCW. Identifying the contribution of mindfulness on QOL above and beyond the effect of other sociodemographic and SCI-related (mainly, pain and functional limitation) factors may guide researchers in developing cost-effective and sustainable mindfulness-based psychosocial interventions in this specific group. Thus, this study aimed to identify the direct and indirect role of mindfulness in the QOL of PwSCI. Based on the previous literature, we hypothesized:

1. Mindfulness predicts high QOL controlling for the effect of sociodemographic and SCI-related variables.
2. Mindfulness, pain, and functional limitation all have unique effects on QOL.
3. The effect of mindfulness on functional limitation is partially mediated by pain.
4. The effect of pain on QOL is partially mediated by functional limitation.
5. The effect of mindfulness on QOL is serially mediated by pain and functional limitation.

## Method

### Procedure

The Institutional Review Board at the University of Wisconsin-Madison approved the study. The researchers collaborated with three leading SCI organizations in the US: the United Spinal Association, North American SCI Consortium, and Paralyzed Veterans of America, for data collection. Each of these organizations posted the study announcement and flyer with a survey link on their websites and social media. Data were collected via an online Qualtrics survey in February and March of 2021. The electronic consent form was provided, and the participants were asked to read and endorse the informed consent form in order to proceed with the survey. In addition, participants had to complete an eligibility questionnaire to fill out the survey. The first 200 participants were provided a $5 Amazon gift card for their participation. Among 251 individuals with SCI who responded to the survey, 13 participants did not complete it. Seven responses were removed due to failure to meet the assumptions of the analysis (i.e., univariate and multivariate outliers). The final sample for this study included 231 participants with SCI who were 18 years or older and did not have a traumatic brain injury that could impair their ability to respond to the questions.

### Measures

#### Sociodemographic and SCI-related questionnaire

Participants’ sociodemographic and SCI-related information was collected through a series of questions developed by the researchers. Participants were asked to provide sociodemographic information, including age, gender identity, race/ethnicity, marital status, educational level, and employment status. The injury-related questionnaires consisted of self-reported information on the level of injury, completeness of injury, cause of injury, time since injury, presence of comorbidities (e.g., chronic physical illness, mental illness, and substance use), and frequency of hospitalization within a year.

### Pain intensity

The intensity of pain was measured using the three-item PROMIS-Pain Intensity short form (3a) questionnaire. The first two items ask individuals to rate their pain intensity within the last seven days, and the third item asks individuals to rate their current pain intensity. Each item is rated on a five-point Likert scale, ranging from 1 (*had no pain*) to 5 (*very severe*). Total scores range from three to 15 and higher scores indicates higher pain intensity [38]. The internal consistency reliability of the scale was 0.41 in the present study. Measurement items in some self-report measures are best conceptualized as causal indicators, where high correlations among items are not expected, and a low alpha coefficient is not an argument against the measure’s validity [39]. Pain levels usually vary from time to time, so items assessing pain in various time frames may be relatively weakly correlated. However, in aggregate, they still provide an index of overall perceived pain levels.

### Functional limitation

Functional limitation was measured using the Self-Report Functional Measure (SRFM) [40]. The SRFM is a modified version of the original Functional Independence Measure, which is the most common functional assessment measure in clinical rehabilitation settings [41]. The SRFM consists of 13 items to measure 13 different motor functions in terms of basic activities of daily living (e.g., “how much help do you need to transfer to and from your bed or chair?“). Participants’ responses are rated on four levels, which range from 1 (*no extra time of help*) to 4 (*total help or never do*). Total scores range from 13 to 52 and higher scores indicate higher functional dependence or limitation. Hoenig et al. [40] reported a high internal consistency with a Cronbach’s alpha coefficient of 0.96, test-retest reliability with most of the kappa coefficients above 0.70, and an intraclass correlation of 0.90. In the present study, the Cronbach’s alpha of the SRFM was 0.93.

### Mindfulness

The Mindful Attention Awareness Scale (MAAS) was used to assess mindfulness in this study. The MAAS is a 15-item instrument that measures mindfulness, with each item rated on a scale from 1 (*almost always*) to 6 (*almost never*) [25]. The MAAS measures the presence or absence of attention to and awareness of things happening in the present moment. The items cover cognitive, emotional, physical, interpersonal, and general domains and reflect the opposite of the construct of mindfulness (i.e., mindlessness). Endorsing the item at a lower frequency represents a higher level of mindfulness (e.g., “I could be experiencing some emotion and not be conscious of it until sometime later.“). These items are worded in such a way because indirect items are more likely to capture one’s state of mindfulness than direct measures of mindfulness [25]. In question 4, the term “walk” was replaced with “move” to fit with this study population, as many PwSCI are unable to walk. Total scores range from 15 to 90 and higher scores indicate greater mindfulness. The previous studies reported good validity and reliability of the MAAS in different populations [25, 42, 43]. The Cronbach’s alpha coefficient of the MAAS in the present study was 0.78.

### Quality of life

Quality of life was measured by the World Health Organization Quality of Life- BREF (WHO-QOL-BREF). The WHOQOL-BREF is a short version of the larger WHOQOL instrument and is a comprehensive self-report QOL measure consisting of 26 items [3]. It measures four domains of QOL with seven items for physical health (e.g., “do you have enough energy for everyday life?“), six items for psychological health (e.g., “how much do you enjoy life?“), three items for social relationship (e.g., “how satisfied are you with your personal relationships?“), eight items for the environment (e.g., “how healthy is your physical environment?“), and two extra items scoring overall perception of QOL and health. Participants rated their responses on a five-point Likert scale. Total scores range from 26 to 130 where higher scores reflect higher QOL. The WHOQOL-BREF has been widely used in different populations and has been reported to be a sound and cross-culturally valid assessment of QOL [44, 45]. In the present study, Cronbach’s alpha coefficient for the WHOQOL-BREF was 0.79.

### Data Analysis

Data analysis was conducted using the Statistical Package for Social Sciences (SPSS) and the R statistical analysis software. Missing data for the variables of interest in the study was less than 3%, and the Expectation-Maximization (EM) algorithm was used in SPSS to impute missing data. Time since injury was excluded in the analysis because the variable had more than 10% of missing data with a non-normal distribution. The multivariate outliers of the data were examined using the Mahalanobis distance test [46] in SPSS. Four multivariate outliers were identified and removed from the final analysis. Data met the assumptions of normality, linearity, homoscedasticity, and multicollinearity.

Means and standard deviations were computed for all continuous study variables (Table 1). Internal consistency reliabilities for each instrument were determined using Cronbach’s alpha coefficients. Bivariate correlations were computed to test associations among continuous variables. The categorical variables were dummy coded for the hierarchical regression analysis. For sociodemographic variables, females and transgender, non-white, single, widowed and divorced, unemployed, and education below bachelor’s degree were coded 0. Male, white, married and cohabitating, employed (part-time, full-time, and self-employed), and bachelor’s degree and above education were coded 1. For injury-related variables, paraplegia, incomplete injury, and absence of comorbidities were coded 1, and tetraplegia, complete injury, and presence of comorbidities were coded 0.

**Table 1:** Descriptive Statistics and Correlations Matrix of Continuous Study Variables

Variables	Mean (SD)	Range	1	2	3	4	5
1.Age (years)	35.60 (8.73)	22–72	1				
2.Hospitalization	1.76 (1.74)	0–6	− 0.42**	1			
3.Pain	8.12 (1.76)	3–12	− 0.27**	0.19*	1		
4.Functional limitation	26.70 (8.73)	13–51	− 0.33**	0.24**	0.55**	1	
5.Mindfulness	55.62 (8.98)	37–79	0.48**	− 0.23**	− 0.42**	− 0.46**	1
6.Quality of life	82.56 (9.70)	58–111	0.47**	− 0.26**	− 0.49**	− 0.46**	0.68**

Note: *p <. 01; **p <. 001

A hierarchical regression analysis was performed to identify the contribution of mindfulness on QOL controlling for the sociodemographic and injury-related variables. Sociodemographic variables: age, gender, race, marital status, education, and employment status, were entered into the first step of the hierarchical model. Injury-related variables: level of injury, completeness of injury, comorbidities, frequency of hospitalization, pain intensity, and functional limitation, were entered into the second step of the model. Finally, mindfulness was entered into the third step. In addition, a serial mediation analysis was conducted to test the indirect effect of mindfulness on QOL through pain and functional limitation. The lavaan package for the R program was used to perform the mediation analysis. The indirect effect was computed using a bootstrap test with 5000 samples and generated bias-corrected 95% confidence intervals.

## Results

### Participant characteristics

The mean age of 231 participants was 35.60 (*SD* = 8.73) years, with a range from 22 to 72 years old (Table 1). Regarding the level of injury, 59.3% of participants had tetraplegia (C1-C8), and 40.7% had paraplegia (T1-S5). The average duration of injury for the 201 participants who disclosed it was 6.41 (*SD* = 7.47) years, ranging from 1 year to 44 years. Nearly 3% of the participants reported the presence of other comorbidities such as hyponatremia, chronic wound, bi-lateral transtibial amputation, and gulf war syndrome. The average frequency of hospitalization within the past year was 1.76 times (*SD* = 1.74). Other sociodemographic and injury-related characteristics of the participants are presented in Table 2.

**Table 2:** Descriptive Statistics of Sociodemographic and Injury-related Information (*N* = 231)

Variables	Categories	*n*	%
Gender identity	Male Female Other	145 85 1	62.8 36.8 0.4
Race/Ethnicity	Caucasian (White) African American Hispanic/Latinx Asian American Native American/American Indian	205 15 5 3 3	88.7 6.5 2.2 1.3 1.3
Marital status	Single Cohabitating Married Windowed/divorced	34 28 149 20	14.7 12.1 64.5 8.7
Education level	Secondary education High school or GED Some college Associate degree Bachelor’s degree Master’s or Doctorate degree	9 60 66 28 51 17	3.9 26.0 28.6 12.1 22.1 7.3
Employment status	Full-time employee (> 30 h per week) Part-time employee (< 30 h per week) Unemployed Retired Self-employed	78 89 46 15 3	33.8 38.5 19.9 6.5 1.3
Level of injury	Cervical Thoracic Lumber Sacral	137 67 18 9	59.3 29.0 7.8 3.9
Completeness of injury	Complete Incomplete	171 60	74.0 26.0
Cause of injury	Motor vehicle accident Fall Sports/physical activity Violent act (e.g., gunshot, explosion) Others	114 51 47 12 7	49.4 22.1 20.3 5.2 3.0
Other diseases or co-morbidities	Chronic illness Mental illness Alcohol and drug use Others Chronic and mental illness Mental illness and alcohol use None	35 10 2 6 10 3 165	15.2 4.3 0.9 2.6 4.3 1.3 71.4

### Correlation

All the continuous study variables were significantly correlated with each other (Table 1). The QOL was positively correlated with age and mindfulness and negatively correlated with the frequency of hospitalization, pain, and functional limitation. Mindfulness was positively associated with age and negatively related to the frequency of hospitalization, pain, and functional limitation.

### Hierarchical regression

In the hierarchical regression analysis, the sociodemographic variables accounted for 36% of the variance in QOL (*R*^2^ = 0.36, *F*_(6, 224)_ = 0.20.76, *p* < .001). The addition of injury-related variables in the second model yielded a 13% increase in the variance explained in QOL (*R*^2^ = 0.49, Δ*R*^2^ = 0.13, Δ*F*_(6, 218)_ = 9.62, *p* < .001). Mindfulness entered in the third model accounted for an additional 10% variance in QOL (*R* = .59, Δ*R*^2^ = 0.10, Δ*F*_(1, 217)_ = 55.18, *p* < .001). Even after controlling for sociodemographic and injury-related factors, mindfulness significantly contributed to high QOL in PwSCI (Table 3).

**Table 3:** Hierarchical Regression Analysis Predicting Quality of Life

	Model 1			Model 2			Model 3				
Variables	B	*β*	*p-*value	B	*β*	*p-*value	B	*β*	*p*-value	95% CIs for B	
**Sociodemographic**											
Age	0.58	0.52	< 0.001	0.37	0.33	< 0.001	0.18	0.16	0.007	[0.05,	0.31]
Gender (Female = 0)	3.03	0.15	0.01	1.48	0.07	0.17	0.70	0.04	0.47	[-1.23,	2.63]
Race (Non-white = 0)	− 0.33	− 0.02	0.78	− 0.18	− 0.009	0.86	− 0.37	− 0.02	0.69	[-2.25,	1.50]
Marital status (Single, divorced, widowed = 0)	-1.67	− 0.07	0.27	− 0.30	− 0.01	0.83	-2.1	− 0.09	0.09	[-4.69,	0.35]
Education (Below bachelor = 0)	-1.95	− 0.10	0.07	0.39	0.02	0.73	0.35	0.02	0.73	[-1.69,	2.39]
Employment (Unemployed = 0)	6.53	0.30	< 0.001	4.65	0.21	0.003	4.13	0.18	0.003	[1.38,	6.88]
**SCI-related**											
Level of injury (Tetraplegia = 0)				2.37	0.12	0.04	2.14	0.10	0.04	[0.08,	4.21]
Completeness (Complete = 0)				0.49	0.02	0.70	1.22	0.06	0.29	[-1.07,	3.50]
Comorbidities (Yes = 0)				5.27	0.25	< 0.001	3.33	0.16	0.002	[1.23,	5.44]
Hospitalization				− 0.69	− 0.13	0.04	− 0.41	− 0.07	0.19	[-1.04,	0.22]
Pain				-1.30	− 0.24	< 0.001	− 0.96	− 0.18	0.002	[-1.58,	− 0.35]
Functional limitation				− 0.04	− 0.04	0.58	− 0.01	− 0.01	0.86	[-0.15,	0.13]
**Psychological**											
Mindfulness							0.47	0.44	< 0.001	[0.35	0.60]
*R* ^2^	0.36**			0.49**			0.59**				

Note: *p <. 01; **p <. 001; B = Unstandardized beta coefficient; *β =* Standardized beta coefficient; CIs = Confidence intervals

In the final model, participants’ age, employment status, level of injury, comorbidities, pain, and mindfulness emerged as significant contributors to QOL (Table 3). Older age (*β* = 0.16, *p* = .007), employment (*β* = 0.19, *p* = .003), and absence of comorbidities (*β* = 0.16, *p* = .002) contributed to higher QOL. Individuals with paraplegia were found to have higher QOL than those with tetraplegia (*β* = 0.10, *p* = .04). Pain negatively predicted QOL (*β*=-0.18, *p* = .002). As hypothesized, mindfulness uniquely contributed to QOL (*β* = 0.44, *p* < .001), controlling for the effect of sociodemographic and injury-related variables.

### Mediation analysis

**Fig. 1:**
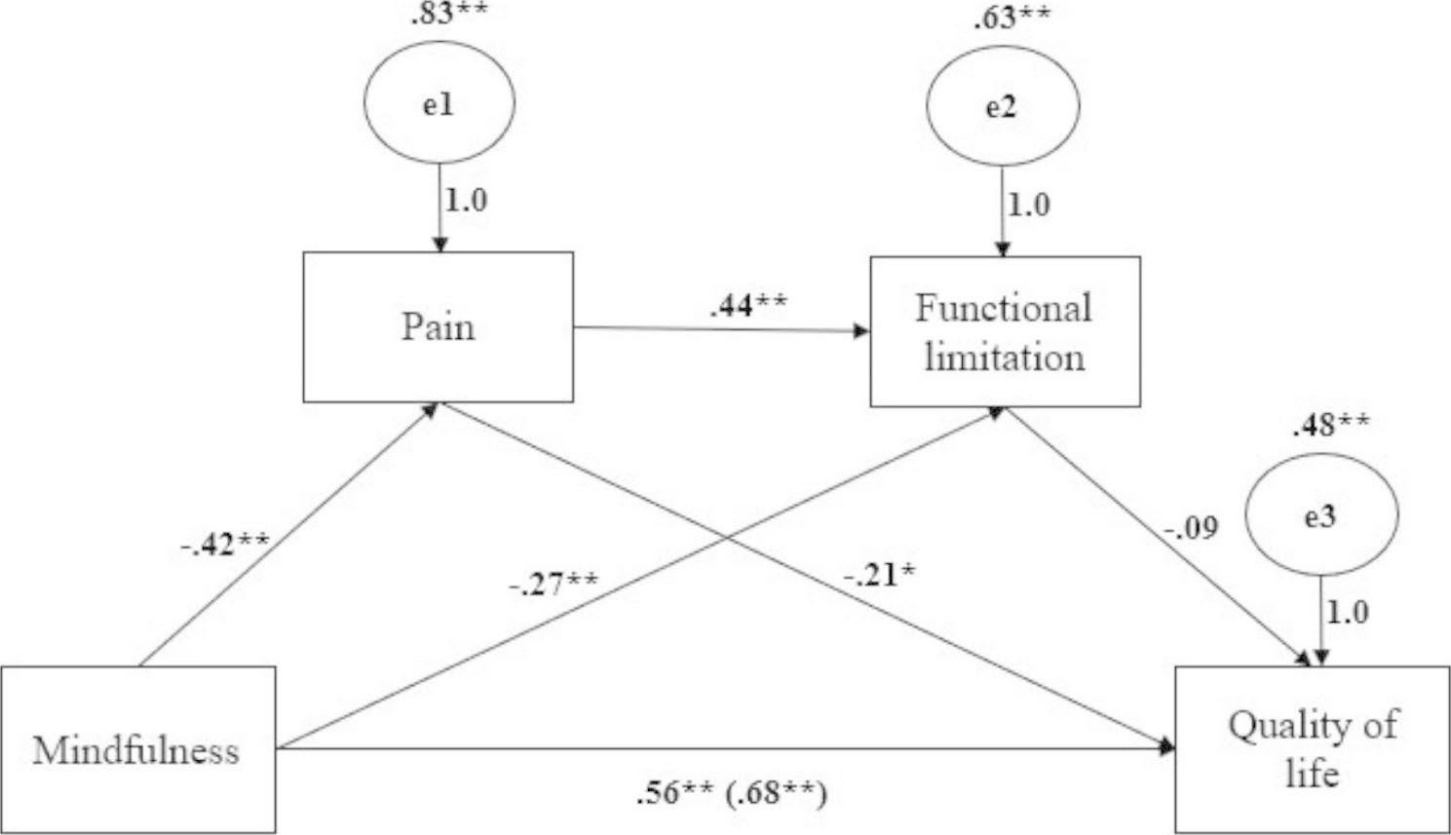
Path coefficients of serial mediation analysis. *p < .01; ** p <.001

The serial mediation analysis was performed to test the direct and indirect effects of mindfulness on QOL (Table 4). There was a significant direct effect of mindfulness on QOL (*β* = 0.56, 95% CI[0.43, 0.68]), controlling for pain and functional limitation (Fig. 1). The pain had a significant direct effect on QOL (*β*=-0.21, 95% CI[-0.36, − 0.06]), and it also mediated the relationship between mindfulness and functional limitation (*β*=-0.18, 95% CI[-0.26, − 0.11]). Because the direct effect of functional limitation on QOL was not significant (*β*=-0.09, 95% CI[-0.24, 0.06]), the mediating effects of functional limitation in the relationship between mindfulness and QOL (*β* = 0.02, 95% CI[-0.02, 0.07]) and pain and QOL (*β*=-0.04, 95% CI[-0.10, 0.03]) were also remained non-significant. The results of the bootstrapping analysis showed that pain and functional limitation did not significantly mediate the relationship between mindfulness and QOL (*β* = 0.02, 95% CI[-0.01, 0.05]). However, the indirect effect of mindfulness on QOL through only pain was significant (*β* = 0.09, 95% CI[0.02, 0.16]). Both mindfulness (*β*=-0.27, 95% CI[-0.40, − 0.14]) and pain (*β* = 0.44, 95% CI[0.29, 0.59]) had significant direct effects on functional limitation. Standardized coefficient values are presented in Fig. 1. Total effect of mindfulness on quality of life is shown in parenthesis.

**Table 4:** Summary of the Indirect Effects on Mediation Analysis

Paths	*β*	*se*	*p*	95% CIs
Mindfulness◊ Pain◊ Functional limitation ◊ QOL	0.02	0.02	0.27	[-0.01, 0.05]
Mindfulness ◊ Pain ◊ QOL	0.09	0.04	0.01	[0.02, 0.16]
Mindfulness ◊ Functional limitation ◊ QOL	0.02	0.02	0.31	[-0.02, 0.07]
Pain ◊ Functional limitation ◊ QOL	− 0.04	0.04	0.28	[-0.10, 0.03]
Mindfulness ◊ Pain ◊ Functional limitation	− 0.18	0.04	< 0.001	[-0.26, − 0.11]

Note: se *=* Standard error; CIs = Confidence intervals; QOL = Quality of life

## Discussion

QOL is often considered a major outcome following the adaptation to the disability process [1] and is the goal of many rehabilitation professionals, including rehabilitation counselors and nurses [2]. The present study sought to identify mindfulness’s direct and indirect effects on QOL of PwSCI. As hypothesized, mindfulness significantly contributed to high QOL above and beyond the impact of established sociodemographic and injury-related variables. There have been no similar studies conducted; however, the finding mirrored some previous studies that demonstrated the positive association between mindfulness or mindfulness-based interventions and QOL or wellbeing [25, 26, 31, 33, 34].

The present findings supported that mindfulness indirectly affects QOL and functional limitation through pain. A few previous studies have provided evidence for the effectiveness of mindfulness or mindfulness-based interventions on reducing pain in people with CIDs [27, 35–37]. The present study findings add insights to a better understanding of the role of mindfulness in reducing pain and thereby increasing functional independence and QOL. However, studies determining the utility of mindfulness for reducing pain and increasing functional independence in PwSCI remain limited. Therefore, more rigorous and larger randomized control research is needed to identify the effectiveness of mindfulness in improving biopsychosocial outcomes post-SCI.

In addition to mindfulness, some sociodemographic and injury-related factors contributed to QOL in PwSCI in this study. Consistent with previous studies [10–12], older and employed individuals were found to have higher QOL than those who were younger and unemployed. It is believed that individuals with SCI tend to have a better adaptation to injury and associated complications as they grow older [12]. However, some researchers argue that aging often comes with multiple additional health problems, functional limitations, and secondary conditions, which may lower QOL with aging [4, 6, 9, 47]. Therefore, it is plausible that only older participants who have health issues, complications, and limitations may have lower QOL compared to those who age in a healthier manner [47]. Therefore, further longitudinal studies are needed to explore how health problems, functional limitations, and secondary complications interact with aging to influence QOL. Regarding employment, the present study adds further evidence on the contribution of employment to higher QOL [9, 11–13, 48]. One of the primary goals of the rehabilitation of PwSCI is returning to employment because employment or return to work is associated with positive rehabilitation outcomes [49].

Regarding injury-related variables, the findings showed that tetraplegia, the presence of pain, and comorbidities negatively influence QOL in this group. People with tetraplegic injury are more likely to experience many disability-related problems, activity limitations, and accessibility issues, impacting an individual’s overall life satisfaction [47]. SCI-related secondary complications (e.g., pain) and comorbidities lead to frequent hospitalization in PwSCI [4, 50]. These secondary complications and comorbidities further increase the frequency and duration of hospitalization [50], decreasing the QOL [14, 15, 51]. The present finding regarding the effect of functional limitation on QOL is not consistent with the literature suggesting the functional limitation’s negative impact on QOL [4, 20, 21]. A significant correlation between functional limitation and QOL in the present study explains that other factors in the model (e.g., pain, mindfulness, age, level of injury) might have attenuated the effect of functional limitation on QOL. Pain was a more critical determinant than functional limitation, and when the effect of pain is statistically controlled, the functional limitation was no longer a significant preditor of QOL.

## Limitations

There are a few limitations to consider while interpreting the study results. First, the survey included only persons with SCI from three agencies in the US. An online survey with self-report questionnaires was used to collect data. The potential participants who were not involved in any of these three agencies and did not have internet access were excluded. Also, compared to the individuals with a high level of physical, psychological, and social functioning, those with greater functional limitations, low education, and unemployment are less likely to access or respond to the online survey. Yet, these organizations are leading SCI organizations in the US; therefore, it is assumed that the study represented a large proportion of the SCI population in the US. In addition, the online survey was anonymous, so the findings were less likely to be influenced by social desirability bias. Second, some literature asserts time since the injury also contributes to QOL [10, 52]; however, this variable was excluded in the current analysis. Despite these limitations, the study findings may have potential implications in the rehabilitation field.

## Implications in practice

Improving QOL is one of the overarching goals in SCI rehabilitation [12]. Mindfulness, a predictor of QOL, is an emerging concept in the rehabilitation field. Even though some individuals may have trait mindfulness, mindfulness skills such as being aware, non-judgmental, and letting go of negative thoughts can be cultivated through formal and informal training to improve QOL [25, 33, 43, 53]. Mindfulness-based interventions, including mindfulness-based stress reduction programs, are proven to facilitate present moment awareness and acceptance effectively, thereby decreasing chronic pain, functional limitation, anxiety, depression, and increasing mental health, QOL, and other positive rehabilitation outcomes [26, 29, 54]. Incorporating mindfulness meditation practices such as breath and posture awareness, body awareness, mindful movement (yoga), and mindful walk into the SCI rehabilitation may substantially benefit PwSCI. Moreover, mindfulness practices can be integrated into everyday life.

Chronic pain is found to be a persistent problem that impacts QOL among PwSCI. It reflects the need to provide effective pain management using both pharmacological and non-pharmacological/psychosocial interventions incorporating mindfulness training. Mindfulness helps individuals relax the body and mind, react less negatively to stimuli, and perceive one’s thoughts as mental processes rather than reality, which leads to decreased emotional distress and pain perception and increased activity and participation [37]. In addition, other theory-based psychosocial interventions, including acceptance-based cognitive behavioral therapy, motivational interviewing, relaxation training (e.g., progressive muscle relaxation, biofeedback), and graded exposure, are beneficial to reduce pain perception [55]. Pain not only directly related to functional limitation but also mediated the role of mindfulness in mitigating the functional limitation. Therefore, reducing pain is likely to improve functional independence, which in turns, contributes to the higher employment and community participation in PwSCI.

Other factors, including age, employment, presence of comorbidities, and level of injury, also need to be considered in the rehabilitation process. Some of these factors are amenable to change with interventions. Early vocational rehabilitation services (e.g., on-the-job support and training, assistive technology, and job development and placement) are promising to help PwSCI obtain and maintain competitive employment and return to work [56, 57]. Vocational services provided by the interdisciplinary team, including rehabilitation nurses and counselors, are more likely to result in positive employment outcomes.

The negative effect of comorbidities on QOL underscores the necessity to give significant attention to the prevention and management of such comorbidities (e.g., mental illness, chronic physical illness, substance use) through education and services. If adequate effort has been given to reduce the incidence and impacts of comorbidities and secondary complications, the rate of re-hospitalizations may be minimized [4]. Notably, people with a higher level of injury (tetraplegia) are likely to have a low QOL. Hence, rehabilitation practitioners should offer additional support to individuals with a higher level of injury during inpatient and community rehabilitation. The findings clearly indicate that sociodemographic, injury-related factors and psychological resources (e.g., mindfulness) need to be taken into account while providing rehabilitation services to PwSCI.

## Conclusion

Mindfulness is a positive psychological resource that can potentially improve psychosocial adaptation in PwSCI. The present study suggests that mindfulness has both direct and buffering effects on QOL. Mindfulness seems to mitigate the effect of pain on functional independence and QOL. This suggests the possibility of the effectiveness of mindfulness-based interventions in reducing the impacts of SCI-related factors on the psychosocial adaptation of PwSCI. Because of the nature and chronicity of SCI, PwSCI may have different needs than those with other chronic conditions. Therefore, tailored mindfulness training might be beneficial for this group. High-quality research is needed to determine the utility of mindfulness interventions on promoting QOL in persons living with chronic disabilities such as SCI.

## Data Availability

The datasets used and/or analyzed during the current study are available from the corresponding author on reasonable request.
